# Supplementary material for: Cannabinoid Type 1 Receptor is Undetectable in Rodent and Primate Cerebral Neural Stem Cells but Participates in Radial Neuronal Migration
Source: Int J Mol Sci. 2020 Nov 17;21(22):8657. doi: 10.3390/ijms21228657 (PMC7696736; doi:10.3390/ijms21228657)
Supplement: Supplementary file 1 [file ijms-21-08657-s001.zip › Morozov_Table-1.docx]

**Supplementary Table 1**.

**Morphologic characteristics of the 3D-reconstructed cells from wild type mouse and rhesus macaque embryo neocortex.**

| Cell code (embryo #) | Cell body location | Number of emitted processes / Cell morpho-functional type | Position of centrosome relative to the nucleus | Position of mother centriole | Volume of the nucleus or chromosomes, μm^3^ | Analyzed segment of cytoplasm, μm^3^ | Number of CB_1_R depositions | | Number of CB_1_R depositions per 100 μm^3^ cytoplasm | |
| --- | --- | --- | --- | --- | --- | --- | --- | --- | --- | --- |
|  |  |  |  |  |  |  | Single | Globule | Single | Globule |
| **Mouse, E16** | | | | | | | | | | |
| M1 (#3) | VZ | 0 / Prometaphase | Mitotic spindle | Cytoplasm | 87.0 | 193.0 | 1 | 0 | 0.5 | 0.0 |
| M2 (#3) | VZ | 1 / Prometaphase | Mitotic spindle | Cytoplasm | 112.5 | 205.5 | 2 | 0 | 1.0 | 0.0 |
| M3 (#3) | VZ | 1 / Early prophase† | Apical | Cilium | 179.5 | 139.8 | 0 | 0 | 0.0 | 0.0 |
| **Average for VZ cells ±SEM** | | | | | **126.3±27.6** |  |  |  | **0.5±0.3** | **0.0** |
| M4 (#1) | SVZ | 2 / INT or IVM | Not found | - | 130.3 | 77.9 | 0 | 0 | 0.0 | 0.0 |
| M5 (#1) | SVZ | 2 / INT or IVM | Not found | - | 104.6 | 83.7 | 1 | 0 | 1.2 | 0.0 |
| M6 (#1) | SVZ | 2 / INT or IVM | Not found | - | 90.5 | 54.3 | 0 | 0 | 0.0 | 0.0 |
| M7 (#1) | SVZ | 2 / INT or IVM | Not found | - | 172.0 | 89.4 | 1 | 0 | 1.1 | 0.0 |
| **Average for SVZ cells ±SEM** | | | | | **124.4±17.9** |  |  |  | **0.6±0.3** | **0.0** |
| M8 (#2) | IZ | 2 / Locomotion | Basal | Cytoplasm | 99.3 | 98.7 | 3 | 0 | 3.0 | 0.0 |
| M9 (#2) | IZ | 2 / Locomotion | Basal | Cytoplasm | 79.9 | 57.0 | 3 | 0 | 5.3 | 0.0 |
| M10 (#2) | IZ | 2 / Locomotion | Basal | Cytoplasm | 100.9 | 85.7 | 2 | 0 | 2.3 | 0.0 |
| M11 (#2) | IZ | 2 / Locomotion | Basal | Cytoplasm | 142.4 | 95.7 | 4 | 2 | 4.2 | 2.1 |
| M12 (#3) | IZ | 1 / Somal translocation | Basal | Cell membrane | 81.4 | 93.6 | 0 | 0 | 0.0 | 0.0 |
| M13 (#3) | IZ | 1 / Somal translocation | Basal | Cell membrane | 78.0 | 69.3 | 0 | 0 | 0.0 | 0.0 |
| M14 (#3) | IZ | 3 / Multi-polar migration | Basal | Cell membrane | 114.7 | 139.7 | 13 | 1 | 9.3 | 0.7 |
| M15 (#3) | IZ | 2 / Lateral migration | Lateral | Cell membrane | 138.8 | 141.5 | 13 | 9 | 9.2 | 6.4 |
| **Average for IZ cells ±SEM** | | | | | **104.4±9.1** |  |  |  | **4.2±1.3** | **1.2±0.8** |
| M16 (#3) | CP | 2 / Locomotion | Basal | Cell membrane | 101.3 | 97.8 | 0 | 0 | 0.0 | 0.0 |
| M17 (#3) | CP | 2 / Locomotion | Basal | Cytoplasm | 106.6 | 135.7 | 0 | 0 | 0.0 | 0.0 |
| M18 (#3) | CP | 2 / Locomotion | Not found | - | 105.4 | 89.9 | 0 | 0 | 0.0 | 0.0 |
| M19 (#3) | CP | 2 / Locomotion | Not found | - | 109.3 | 65.0 | 0 | 0 | 0.0 | 0.0 |
| M20 (#3) | CP | 2 / Locomotion | Not found | - | 112.4 | 81.4 | 0 | 0 | 0.0 | 0.0 |
| M21 (#3) | CP | 2 / Locomotion | Not found | - | 110.4 | 68.0 | 0 | 0 | 0.0 | 0.0 |
| M22 (#3) | CP | 2 / Locomotion | Basal | Cytoplasm | 95.8 | 114.8 | 0 | 0 | 0.0 | 0.0 |
| M23 (#3) | CP | 3 / TVM | Basal | Cytoplasm | 85.4 | 94.9 | 0 | 0 | 0.0 | 0.0 |
| M24 (#3) | CP | 4 / TVM | Basal | Cell membrane | 114.6 | 109.4 | 2 | 1 | 1.8 | 0.9 |
| M25 (#3) | CP | 3 / TVM | Basal | Cytoplasm | 75.6 | 96.7 | 0 | 0 | 0.0 | 0.0 |
| M26 (#4) | CP | 3 / TVM | Basal | Cell membrane | 125.7 | 144.9 | 8 | 1 | 5.5 | 0.7 |
| M27 (#4) | CP | 2 / Locomotion | Basal | Cytoplasm | 126.6 | 123.4 | 8 | 2 | 6.5 | 1.6 |
| M28 (#4) | CP | 2 / Locomotion | Not found | - | 70.6 | 36.2 | 0 | 0 | 0.0 | 0.0 |
| M29 (#4) | CP | 2 / Locomotion | Basal | Cilial vesicle | 107.9 | 117.4 | 0 | 1 | 0.0 | 0.9 |
| M30 (#4) | CP | 2 / Locomotion | Basal | Cilial vesicle | 95.5 | 96.9 | 2 | 1 | 2.1 | 1.0 |
| **Average for CP cells ±SEM** | | | | | **102.9±4.2** |  |  |  | **1.1±0.6** | **0.3±0.1** |
| M31 (#4) | MZ | 2 / Lateral migration | Aside | Cytoplasm | 77.0 | 64.1 | 3 | 0 | 4.7 | 0.0 |
| M32 (#3) | MZ | 2 / Lateral migration | Lateral | Cytoplasm | 143.9 | 172.9 | 32 | 24 | 18.5 | 13.9 |
| M33 (#3) | MZ | 2 / Lateral migration | Lateral | Cytoplasm | 147.1 | 167.1 | 39 | 23 | 23.3 | 13.8 |
| M34 (#3) | MZ | 2 / Lateral migration | Lateral | Cytoplasm | 101.9 | 149.0 | 5 | 1 | 3.4 | 0.7 |
| **Average for MZ cells ±SEM** | | | | | **117.5±17.0** |  |  |  | **12.5±5.0** | **7.1±3.9** |
| **Rhesus macaque, E45** | | | | | | | | | | |
| R1 (#1) | VZ | 1 / Early prophase† | Apical | Cilium | 124.5 | 60.5 | 4 | 0 | 6.6 | 0.0 |
| R2 (#1) | VZ | 2 / INT or IVM | Not found | - | 59.5 | 28.5 | 0 | 0 | 0.0 | 0.0 |
| R3 (#1) | VZ | 2 / INT or IVM | Not found | - | 54.1 | 30.4 | 1 | 0 | 3.3 | 0.0 |
| R4 (#1) | VZ | 2 / INT or IVM | Not found | - | 66.6 | 30.4 | 0 | 0 | 0.0 | 0.0 |
| R5 (#1) | VZ | 2 / INT or IVM | Not found | - | 61.7 | 20.8 | 0 | 0 | 0.0 | 0.0 |
| R6 (#1) | VZ | 2 / INT or IVM | Not found | - | 55.7 | 20.3 | 0 | 0 | 0.0 | 0.0 |
| **Average for VZ cells ±SEM** | | | | | **70.4±11.0** |  |  |  | **1.7±1.1** | **0.0** |
| R7 (#2) | SVZ | 2 / INT or IVM | Not found | - | 78.9 | 34.9 | 1 | 0 | 2.9 | 0.0 |
| R8 (#2) | SVZ | 2 / IVM | Apical | Cilium | 64.5 | 45.2 | 1 | 0 | 2.2 | 0.0 |
| R9 (#2) | SVZ | Truncated / IVM‡ | Apical | Cell membrane | - | - | - | - | - | - |
| R10 (#2) | SVZ | 1 / INT or IVM | Not found | - | 71.9 | 28.2 | 0 | 0 | 0.0 | 0.0 |
| R11 (#2) | SVZ | 2 / INT or IVM | Not found | - | 72.4 | 29.4 | 0 | 0 | 0.0 | 0.0 |
| R12 (#2) | SVZ | 2 / INT or IVM | Not found | - | 70.3 | 34.6 | 1 | 0 | 2.9 | 0.0 |
| R13 (#2) | SVZ | 2 / INT or IVM | Not found | - | 73.8 | 32.8 | 1 | 0 | 3.0 | 0.0 |
| R14 (#2) | SVZ | 2 / INT or IVM | Not found | - | 86.9 | 43.2 | 1 | 0 | 2.3 | 0.0 |
| R15 (#2) | SVZ | 2 / INT or IVM | Not found | - | 112.6 | 34.0 | 1 | 0 | 2.9 | 0.0 |
| R16 (#2) | SVZ | 2 / INT or IVM | Not found | - | 116.9 | 38.6 | 0 | 0 | 0.0 | 0.0 |
| R17 (#2) | SVZ | 2 / INT or IVM | Not found | - | 83.1 | 36.8 | 3 | 0 | 8.2 | 0.0 |
| **Average for SVZ cells ±SEM** | | | | | **83.1±5.7** |  |  |  | **2.4±0.8** | **0.0** |
| R18 (#2) | IZ | 1 / Somal translocation | Aside | Cell membrane | 92.8 | 53.2 | 0 | 0 | 0.0 | 0.0 |
| R19 (#2) | IZ | 2 / Locomotion | Not found | - | 72.0 | 27.6 | 0 | 0 | 0.0 | 0.0 |
| R20 (#2) | IZ | 2 / Locomotion | Basal | Cell membrane | 90.8 | 65.6 | 0 | 0 | 0.0 | 0.0 |
| R21 (#1) | IZ | 2 / Locomotion | Basal | Cell membrane | 88.8 | 54.7 | 16 | 4 | 29.3 | 7.3 |
| R22 (#1) | IZ | 2 / Locomotion | Basal | Cytoplasm | 99.9 | 72.1 | 5 | 7 | 6.9 | 9.7 |
| R23 (#1) | IZ | 2 / Locomotion | Aside | Cytoplasm | 97.9 | 61.4 | 11 | 5 | 17.9 | 8.1 |
| **Average for IZ cells ±SEM** | | | | | **90.4±4.1** |  |  |  | **9.2±5.0** | **4.2±1.9** |
| R24 (#1) | CP | 2 / Locomotion | Aside | Cell membrane | 112.2 | 102.7 | 14 | 0 | 13.6 | 0.0 |
| R25 (#1) | CP | 2 / Locomotion | Basal | Cell membrane | 119.0 | 79.2 | 2 | 0 | 2.5 | 0.0 |
| R26 (#2) | CP | 3 / TVM | Basal | Cell membrane | 111.9 | 135.7 | 53 | 5 | 39.1 | 3.7 |
| R27 (#2) | CP | 2 / Locomotion | Basal | Cell membrane | 90.4 | 93.0 | 38 | 14 | 40.9 | 15.1 |
| R28 (#2) | CP | 4 / TVM | Basal | Cytoplasm | 106.6 | 124.9 | 82 | 14 | 65.7 | 11.2 |
| R29 (#1) | CP | 2 / Locomotion | Basal | Cytoplasm | 86.5 | 78.5 | 18 | 4 | 22.9 | 5.1 |
| R30 (#1) | CP | 3 / TVM | Basal | Cytoplasm | 97.4 | 89.8 | 19 | 3 | 21.2 | 3.3 |
| R31 (#1) | CP | 2 / Locomotion | Basal | Cytoplasm | 90.6 | 95.8 | 34 | 9 | 35.5 | 9.4 |
| R32 (#1) | CP | 2 / Locomotion | Aside | Cell membrane | 87.4 | 86.5 | 26 | 6 | 30.1 | 6.9 |
| **Average for CP cells ±SEM** | | | | | **100.2±4.1** |  |  |  | **30.2±6.1** | **6.1±1.7** |
| R33 (#1) | MZ | 2 / Lateral migration | Lateral | Cell membrane | 111.0 | 76.0 | 28 | 7 | 36.8 | 9.2 |
| R34 (#2) | MZ | 2 / Lateral migration | Lateral | Cilial vesicle | 100.6 | 83.4 | 52 | 8 | 62.4 | 9.6 |
| **Average for MZ cells ±SEM** | | | | | **105.8±5.2** |  |  |  | **49.6±12.8** | **9.4±0.2** |

† - Centrosome is duplicated for initiation of mitosis.

‡ - This incompletely reconstructed cell was arbitrarily chosen for 3D reconstruction and excluded from quantifications of random cells.

Single – small depositions of DAB-Ni immunoprecipitations in cytoplasm; Globule – conglomerates of DAB-Ni immunoprecipitations around intracellular vesicles. Abbreviations: INT, interkinetic nuclear translocation; IVM, initial vertical migration; TVM, terminal vertical migration.
