# Supplementary material for: Cannabinoid Type 1 Receptor is Undetectable in Rodent and Primate Cerebral Neural Stem Cells but Participates in Radial Neuronal Migration
Source: Int J Mol Sci. 2020 Nov 17;21(22):8657. doi: 10.3390/ijms21228657 (PMC7696736; doi:10.3390/ijms21228657)
Supplement: Supplementary file 1 [file ijms-21-08657-s001.zip › Morozov_Table-2.docx]

**Supplementary Table 2**.

**Morphologic characteristics of the 3D-reconstructed cells from E14 CB_1_R^-/-^ mouse embryo neocortex.**

| Cell code (embryo #) | Cell body location | Number of emitted processes / Cell morpho-functional type | Position of centrosome relative to the nucleus | Position of mother centriole | Volume of the nucleus, μm^3^ | Analyzed segment of cytoplasm, μm^3^ | Number of CB_1_R depositions | | Number of CB_1_R depositions per 100 μm^3^ cytoplasm | |
| --- | --- | --- | --- | --- | --- | --- | --- | --- | --- | --- |
|  |  |  |  |  |  |  | Single | Globule | Single | Globule |
| KO1 (#1) | IZ | 4 / Multi-polar migration | Basal | Cytoplasm | 68.0 | 59.4 | 3 | 0 | 5.1 | 0.0 |
| KO2 (#1) | IZ | 8 / Multi-polar migration | Basal | Cilial vesicle | 71.6 | 62.8 | 1 | 0 | 1.6 | 0.0 |
| KO3 (#1) | IZ | 2 / Vertical; early prophase† | Basal | Cell membrane | 77.6 | 55.4 | 3 | 0 | 5.4 | 0.0 |
| KO4 (#1) | IZ | 3 / Multi-polar migration | Basal | Cilial vesicle | 66.0 | 57.8 | 0 | 0 | 0.0 | 0.0 |
| KO5 (#1) | IZ | 7 / Multi-polar migration | Basal | Cytoplasm | 59.6 | 54.8 | 1 | 0 | 1.8 | 0.0 |
| KO6 (#1) | IZ | 8 / Multi-polar migration | Basal | Cilial vesicle | 70.8 | 60.4 | 0 | 0 | 0.0 | 0.0 |
| KO7 (#1) | IZ | 1 / INT or IVM | Not found | - | 62.8 | 30.2 | 1 | 0 | 3.3 | 0.0 |
| KO8 (#2) | IZ | 4 / Multi-polar migration | Not found | - | 82.0 | 50.2 | 0 | 0 | 0.0 | 0.0 |
| KO9 (#2) | IZ | 2 / Locomotion | Aside | Cilial vesicle | 97.6 | 99.8 | 3 | 0 | 3.0 | 0.0 |
| KO10 (#2) | IZ | 2 / Locomotion | Basal | Cytoplasm | 96.8 | 85.2 | 0 | 0 | 0.0 | 0.0 |
| KO11 (#2) | IZ | 8 / Multi-polar migration | Aside | Cilial vesicle | 67.6 | 64.9 | 1 | 0 | 1.5 | 0.0 |
| KO12 (#2) | IZ | 3 / Multi-polar migration | Basal | Cytoplasm | 84.0 | 71.8 | 1 | 0 | 1.4 | 0.0 |
| KO13 (#3) | CP | 1 / Somal translocation | Not found | - | 117.2 | 75.0 | 0 | 0 | 0.0 | 0.0 |
| KO14 (#3) | CP | 2 / Locomotion | Not found | - | 119.6 | 82.8 | 0 | 0 | 0.0 | 0.0 |
| KO15 (#3) | CP | 2 / Locomotion | Not found | - | 90.4 | 48.2 | 0 | 0 | 0.0 | 0.0 |
| KO16 (#3) | CP | 2 / Locomotion | Not found | - | 112.8 | 64.2 | 2 | 0 | 3.1 | 0.0 |
| **Average for CB_1_R^-/-^ mouse cells ±SEM** | | | | | **84.0±4.9** |  |  |  | **1.6±0.5** | **0.0** |

† - Centrosome is duplicated for initiation of mitosis that reorganize the cytoskeleton and interrupts the cell migration.

Single – small depositions of DAB-Ni immunoprecipitations in cytoplasm; Globule – conglomerates of DAB-Ni immunoprecipitations around intracellular vesicles. Abbreviations: INT, interkinetic nuclear translocation; IVM, initial vertical migration.
